# Nanodrugs and Natural Pharmaceutical Products

**Published:** 2012-05-28

**Authors:** Mohammadreza Abbaspour

**Affiliations:** 1 Nanotechnology Research Center and School of Pharmacy, Ahvaz Jundishapur University of Medical Sciences, Ahvaz, IR Iran

**Keywords:** Nanotechnology, Pharmacology

Jundishapur University of Medical Sciences (AJUMS) officiated as host to the second nanodrugs congress on March 2012, which was held by nanotechnology research center of AJUMS in cooperation with Shahid Chamran University of Ahvaz, Iran. This scientific event was scheduled to be held in a bid to create an appropriate environment for idea exchange among the specialists and researchers active in the field of drug delivery. The objectives of the organization of this congress was introducing the latest achievements in the field of nanodrugs, considering targeted drug delivery aim for the treatment of incurable diseases, improving the life quality and social health, and converting science and technology into wealth.

One of the major scopes of the congress was the application of nanotechnology in the field of natural pharmaceutical products. Recently there have been considerable researches on developing biocompatible and biodegradable nanocarriers/nanodevices as novel drug delivery systems. Natural polymers or biopolymers are generally biocompatible, biodegradable, non-toxic and non-immunogenic. They occur widely in nature and are classified into 2 groups; polysaccharides and proteins (1). Chitosan, starch, dextran, and alginate are examples of commonly used polysaccharides while collagen, gelatin, and albumin are examples of commonly used proteins. These biopolymers are widely applied in formulation of nanospheres, nanocapsules, and recently nanofibers in order to enhance drug delivery to specific pharmacological sites or tissue engineering.

Lipids also are a broad group of naturally occurring molecules that include fats, waxes, sterols, phospholipids, fat-soluble vitamins, mono-, di-, and tri-glycerides which are advantageous to formulation of a wide range of lipid-based nanocarriers; such as solid lipid nanoparticles (SLN), nanostructured lipid carriers (NLC), and lipid drug conjugates (LDC) to minimize the drawbacks associated with polymeric nanoparticles specially low drug loading for hydrophobic drugs (2).

Moreover some research are being focused on development of novel drug delivery systems for herbal extracts or plant actives, such as polymeric nanoparticles, nanocapsules, liposomes, phytosomes, nanoemulsions, microsphere, transferosomes, and ethosomes, to protect active herbal ingredients from physical and chemical degradation, enhance safety and pharmacological activity, and overcome solubility and bioavailability problems associated with plant medicine (3). Finally, future prospects of advancements in utilizing nanotechnology for drug delivery, cancer therapy, gene therapy, tissue engineering etc. represents the need to further efforts and focus of researchers in this field.
